# Teaching heuristics and mnemonics to improve generation of differential diagnoses

**DOI:** 10.1080/10872981.2020.1742967

**Published:** 2020-03-17

**Authors:** F. Stuart Leeds, Kareem M. Atwa, Alexander M. Cook, Katharine A. Conway, Timothy N. Crawford

**Affiliations:** a Family Medicine, Wright State University Boonshoft School of Medicine (WSUBSOM), Fairborn, OH, USA; b PGY-1 Resident, Bethesda Hospital Family Medicine Residency Program, Cincinnati, OH, USA; c PGY-1 Resident, University of Chicago Family Medicine Residency Program, Chicago, IL, USA; d Population and Public Health Sciences, Wright State University Boonshoft School of Medicine (WSUBSOM), Fairborn, OH, USA

**Keywords:** Differential diagnosis, heuristics, mnemonics, metamemory, predoctoral education

## Abstract

**Background**: Differential diagnosis (DDx) is one of the key cognitive skills that medical learners must develop. However, little is known regarding the best methods for teaching DDx skills. As metacognition plays a fundamental role in the diagnostic process, we hypothesized that the teaching of specific heuristics and mnemonics collectively termed metamemory techniques (MMTs) would enhance the capacity of medical students to generate differential diagnoses.

**Methods**: In a 90-min DDx workshop, third-year medical students (N = 114) were asked to generate differentials before and after learning each of four MMTs. Differential sizes were compared using a linear mixed-effect model. Students also completed a post-session questionnaire which included a subjective ranking of the MMTs, as well as Likert-scale and free-text sections for course feedback.

**Results**: One MMT (the *Mental CT Scan*, an anatomic visualization technique) significantly increased the size of student differentials (+13.3%, p =.0005). However, a marked cumulative increase across all four MMTs was noted (+36.5%, p <.0001). A majority of students ranked the *Mental CT Scan* the most useful MMT (51.5%). They found the workshop both worthwhile (4.51/5, CI 4.33–4.69) and enjoyable (4.33/5, CI 4.12–4.55), and considered the MMTs they learned useful and practical (4.49/5, CI 4.32–4.67).

**Conclusion**: The MMT-based DDx workshop was effective in enhancing the skill of DDx generation, and was rated very favorably by students.

## Introduction

Differential diagnosis (DDx), the cognitive process of producing and prioritizing a list of potential diagnoses for a given clinical presentation, is one of the most important and difficult skills a medical student (or indeed, a clinician at any level of experience) must develop. The student begins to learn the rudiments of forming differentials as early as the first year of medical school, and proceeds in earnest to further advance those skills with actual patients ‘on the wards’ – but in truth, DDx is a skill set that requires a lifetime to master. Despite the centrality of DDx in a physician’s clinical ‘toolbox,’ the available literature gives little guidance as to the most effective way to teach DDx to medical students. Similarly, while it may be that some US medical schools include DDx as a discrete subject, few have published reports describing such curricula – suggesting that at least some schools still prefer the traditional, more indirect and inferential approach of case studies and hands-on clinical experience [1,2]. This may, in part, be the result of the limited studies that describe or propose overt DDx teaching methodologies, many of which have produced disappointing results. Courses that simply teach abstract clinical reasoning skills, or Bayesian competing-hypothesis approaches have not been shown to enhance students’ diagnostic skill [3,4] and perhaps this is not surprising, given that experienced physicians do not appear to undertake the process of diagnosis in this overtly analytical manner [1,5,6].

What is needed, then, is a more grounded, pragmatic approach to DDx that reflects what working physicians are actually doing – or should be doing – on a day-to-day basis. Clinicians generally employ a mixture of analytic and nonanalytic approaches to the diagnostic process [7–9], and crucially, there is a metacognitive component that is essential but frequently overlooked [1,10,11]; in other words, awareness of and access to one’s own knowledge about the subject at hand is, arguably, as important as the knowledge itself. Metacognition encompasses a broad range of processes involved in the control and monitoring of thinking and learning [8,12–14]. Pursuant to that, we have developed a model for conceptualizing and teaching DDx that is streamlined and practical, while accounting for the key role of metacognition, and in particular, of *metamemory*, the conscious methods and mechanisms for conducting memory operations. The model separates the process of DDx into three sequential (though necessarily overlapping) components:

1. *Generation –* populating a large, inclusive list of possible diagnoses
2. *Filtration* – removing diagnoses that do not fit the clinical dataset
3. *Ordering –* ranking the diagnoses (in terms of both likelihood and risk to the patient)

This bears some similarity to, but is considerably simpler than other proposed DDx models, such as the Identify-Frame-Organize-Limit-Explore-Rank-Test paradigm described by Stern et al. [15].

In our experience, and as noted elsewhere [16–18], generation seems to be a ‘rate-limiting step’ for most medical students. And clearly, a failure to generate a robust candidate list of diagnoses will render moot the processes of filtration and ordering. Though there is considerable literature addressing the heuristics and biases that result in diagnostic error [6,8,13,19,20], the generative process might be considered anterior to most of these, since a diagnostician cannot entertain, let alone dismiss or overlook a potential diagnosis that hasn’t even been raised as a possibility [21,22]. Therefore, our primary focus in this study is to understand what may influence and enhance a student’s capacity for DDx generation.

The generation problem may not be primarily one of knowledge deficit. By the third year of medical school, the average student has acquired an impressive fund of clinical knowledge. But difficulties with DDx may stem in greater part from metacognitive limitations, such that the student struggles with integration, organization, and recall of information [12,14,23]. One straightforward and time-tested approach to this problem is the use of what can broadly be called *metamemory techniques* (MMTs) – mnemonic devices and heuristics (‘mental shortcuts’) that serve to remind students and clinicians what they may already know in another context. Students and educators often think of MMTs as gimmicks or ‘tricks,’ but such devices have been shown to enhance academic performance in a variety of settings [24–26]; unfortunately, little is known about the efficacy of MMTs in medical learning, recall, and reasoning, particularly as applied to student’s development of DDx skills. DDx ‘mnemonics’ (an informal term generally used to refer to a subset of MMTs that includes acrostic and phrase expression devices) may be general-purpose or specific to particular clinical situations, and are deployed widely at all levels of training in medicals schools and residency programs [18,27,28], but there are essentially no studies validating their use or commending their transmission to learners. Moreover, it is unclear as to which MMTs are the most effective in helping students to generate differentials.

In this study, our hypotheses are driven by several related questions:

1. Will the explicit teaching of appropriate MMTs to third-year medical students result in measurable improvements in the size of their differential diagnoses?
2. Which MMTs are the most effective? Which are least effective?
3. Which MMTs do students subjectively find most helpful? Do their impressions correlate with objective assessments of MMT efficacy?

## Methods

### Participants

Third-year Family Medicine (FM) clerkship students (MS3s) at the Wright State University Boonshoft School of Medicine were evaluated over one full school year (N = 114), in eight groups (6-week rotations) of 12–20 students. MS3s were anonymized using a unique identifier consisting of a self-assigned random word + random number, which they were to use on all documents throughout the course of the study.

### Study design

Four DDx MMTs were identified and selected for study, based on their broad applicability, apparent common usage in clinical settings, and the teaching experience of the authors, as well as on the relevant literature [15,17,18,28,29]. These are enumerated in Table 1.

**Table 1.** Metamemory techniques used in the study

*MMT*	Description
Constellations	Grouping subsets of medically-relevant facts to produce mulitple sub-differentials
Mental CT scan	Coronal plane, anterior-to-posterior visualization of tissues and organs for anatomic differentials
VINDICATES	Acrostic device that produces differentials by pathophysiology
Bundling	‘Chunking’ heuristic which leverages awareness that certain diagnoses tend to frequently co-occur in a differential

MS3s, as part of their FM didactics curriculum, participated in a 90-min experimental ‘self-examining classroom’ workshop designed to teach the use of DDx MMTs according to the following protocol:

MMTs were presented in a fixed order, as listed in Table 1. For each MMT:

1. Students were given a clinical case, consisting of a brief history and exam for a fictional patient, and asked to produce an unsorted differential diagnosis within a 3-min time limit. These ‘pre-cases’ (prior to instruction in use of the MMT) were returned to the course proctor.
2. Students were then trained in the use of the MMT, which was simply identified as a DDx ‘trick.’ Training generally included a brief demonstration case and a discussion of the practical application of the MMT.
3. Students then completed and returned a 3 min ‘post-case.’

A crossover control design was employed, such that for each MMT, there were two assay cases: a Case A and B. At the beginning of the session, the class was evenly divided into left and right sides. For the pre-case, the left completed Case A and the right completed case B. For the post-case, the left side completed Case B, and the right completed case A. This approach made it possible to measure the pre/post change in a specific case, without the biases introduced by students working the same case more than once. The same case-pairs were used for each MMT throughout the study, always in the order enumerated in Table 1.

Differential sizes were determined in two ways. Unscreened differential sizes were assessed as a simple tally of the number of diagnoses a student listed for a given case. For screened differentials, the raw lists were parsed by medical readers (AC, KA, FL), and inapplicable diagnoses were removed.

A mandatory post-experience questionnaire was also provided to students (N = 56), beginning with clerkship Rotation 3. This questionnaire included Likert-scale questions related to the course and to pre-course experiences with DDx, and also asked students to rank the 4 MMTs in terms of perceived effectiveness. A free-text optional feedback section was also included. Questionnaires were marked with the anonymous identifiers selected at the start of the course.

### Analysis

Case data were collected in a Microsoft Excel spreadsheet and indexed by anonymous identifier. Differentials for all pre-post cases were scored by fourth-year medical students (AC, KA) for gross size (unscreened), and size of clinically plausible (screened) differentials. Questionnaire data were collected and indexed by anonymous identifier in a separate spreadsheet.

Data were analyzed using SAS version 9.4 (Cary, NC). Descriptive Statistics were conducted with means, standard deviations, and 95% confidence intervals (CI) for all continuous variables. Analysis of Variance (ANOVA) was used to assess differences in unscreened and screened scores. Post-hoc tests were conducted using Tukey’s method. To assess the impact of the differential diagnoses interventions, two linear mixed-effects model (screened and unscreened) was conducted. The models included a group variable (i.e., Case A first or Case B first), iteration [1–8], clerkship rotation, a group-by-iteration interaction, and a rotation-by-iteration interaction. To examine within and between group differences, Tukey’s post hoc tests were conducted to adjust for multiple comparisons. All p-values <.05 were regarded as statistically significant. Graphs were produced using Excel or GraphPad Prism (San Diego, CA).

## Results

A total of 114 Family Medicine clerkship students, divided into 8 sequential 6-week rotations of 12–20 students, participated in the study. Approximate half (n = 56) received the cases in the pre-post intervention order A-B, with the remainder receiving them in order B-A. Over the entire study, the mean unscreened and screened DDx scores (size of differentials) for the groups combined were 8.14 (95% CI = 7.94–8.35) and 6.69 (95% CI = 6.52–6.87), respectively.

### Cumulative and individual effect of MMTs

**Figure 1.**
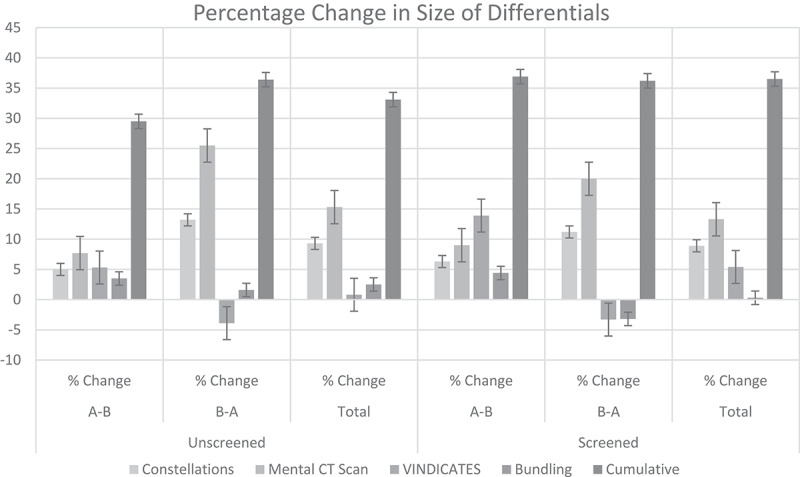
Per-MMT and cumulative change in differential size after training

These results are presented in Table 2 and Figure 1. For the A-B, B-A, and combined groups, the cumulative scores increased by 29.5% (p = .03), 36.4% (p = .0002), and 33.1% (p < .0001), respectively. Of the four MMTs tested, only the *Mental CT Scan* was found to independently increase DDx scores. This trend was observed in both test groups, and the effect was statistically significant for the combined group (A-B + B-A) of students. Results for cumulative scores and the *Mental CT Scan* were similar for screened and unscreened groups.

### Post-experience survey

The quantitative results are summarized in Table 3. In general, students rated the experience as worthwhile, practically useful, and enjoyable. Students did not rate their pre-course DDx skills or awareness of the tested MMTs highly. There was strong agreement that DDx was a key skill to be acquired, and agreement that a dedicated DDx course should be part of the medical school curriculum.

**Table 2.** Changes in differential size after learning MMTs

	Unscreened						Screened					
	A-B		B-A		All		A-B		B-A		All	
MMT	*Diff* *(%)*	*p*	*Diff* *(%)*	*p*	*Diff* *(%)*	*p*	*Diff (%)*	*p*	*Diff (%)*	*p*	*Diff* *(%)*	*p*
Constellations	0.33 (5.0)	.99	0.94 (13.2)	.93	0.64 (9.3)	.16	0.33 (6.3)	.99	0.66 (11.2)	.99	0.50 (8.9)	.41
Mental CT Scan	0.73 (7.7)	.99	1.74 (25.5)	.09	1.24 (15.3)	<.0001	0.71 (9.0)	.97	1.06 (20.0)	.60	0.88 (13.3)	.005
VINDICATES	0.42 (5.3)	.99	−0.30 (−3.9)	.99	0.06 (0.8)	.99	0.89 (13.9)	.85	−0.21 (−3.3)	.99	0.34 (5.4)	.83
Bundling	0.29 (3.5)	.99	0.15 (1.6)	.99	0.22 (2.5)	.99	0.30 (4.4)	.99	−0.27 (−3.2)	.99	0.02 (0.3)	.99
Cumulative	1.94 (29.5)	.03	2.61 (36.4)	.0002	2.27 (33.1)	<.0001	1.94 (36.9)	.003	2.14 (36.2)	.0003	2.04 (36.5)	<.0001

Diff = difference in pre-MMT and post-MMT size of differentials, expressed as absolute and percentages for students who received the A case first (A-B), B case first (B-A), and all cases combined. Unscreened differentials are raw counts of students’ listed diagnoses; screened differential sizes were edited by medical readers (AC, KA)

**Table 3.** Follow-up questionnaire

	Mean (sd)	95% CI
*1. The information presented in the DDx course was worthwhile and valuable*	4.51 (0.74)	4.33–4.69
*2. The techniques presented in the DDx course are useful and practical in real clinical situations*	4.49 (0.72)	4.32–4.67
*3. I found the DDx course enjoyable*	4.33 (0.89)	4.12–4.55
*4. My DDx skills were strong prior to the course*	3.15 (0.98)	2.91–3.38
*5. My DDx skills improved as a result of the course*	4.20 (0.74)	4.03–4.38
*6. I had already learned the presented techniques earlier in my educational experience*	2.84 (1.18)	2.55–3.12
*7. I think DDx is a key skill to develop during medical school*	4.78 (0.62)	4.63–4.93
*8. I think there should be a course (or courses) in medical school dedicated to teaching DDx skills*	4.30 (0.94)	4.08–4.53

In terms of perceived usefulness, students in the survey expressed their preferences for the MMTs in the order

*Mental CT Scan ≫ Constellations > Bundling > VINDICATES*

with more than 50% preferring Mental CT Scan (Table 4).

**Table 4.** DDx MMT ranking (N = 68)

	Constellations	Mental CT Scan	VINDICATES	Bundling
Rank	n (%)	n (%)	n (%)	n (%)
1	14 (20.3)	35 (51.5)	9 (13.2)	11 (16.2)
2	9 (13.0)	19 (27.9)	18 (26.5)	22 (32.3)
3	22 (31.9)	10 (14.7)	16 (23.5)	20 (29.4)
4	34 (34.8)	4 (5.9)	25 (36.8)	15 (22.1)

Free-texted student comments were also collected, and these reflected very positive perceptions of the content and format of the course overall. Critical feedback included a number of recommendations to: a) grant students more time for case completion, b) include opportunities for open discussion of completed cases, and c) shorten the duration of the course.

## Discussion

In this study, we have demonstrated that the teaching of specific MMTs to 3rd-year Family Medicine clerkship students resulted in small but significant improvements in their capacity to generate differential diagnoses. Our specific findings may be summarized as follows:

1. For the total study population, a small but significant increase in the size of both screened and unscreened differentials was observed from the beginning to the end of the course, in a manner suggesting an additive effect contributed by each MMT.
2. The MMT referred to as the *Mental CT Scan* was the only one of the tested techniques that produced an independent, statistically significant positive effect on differential size.
3. Students, consonant with their performance on the test cases, rated the *Mental CT Scan* as the most useful MMT of the four techniques presented.
4. Students rated the DDx course highly in terms of perceived importance, usefulness, and enjoyability, and made valuable recommendations for course improvement.

DDx is one of the essential cognitive skills a clinician must master. A failure to generate adequate and appropriate differentials is a likely root cause of diagnostic error – and such errors frequently put patients at risk. Singh et al. [30] have estimated that, in the US outpatient population alone, perhaps 12 million patients a year are exposed to errors in diagnosis, half of which result in measurable harm. The problem is as serious as it is complex, and it is reasonable to suggest that the solution begins with finding better ways to teach diagnostic skills to students and residents [31]. Yet there is little consensus as to the best way to accomplish this. With few exceptions, textbooks of differential diagnosis do not generally articulate a systematic approach to the DDx process [32], and the material therein is often presented without explicit evidence or citations [27,28,33]. It may be that some medical schools are teaching DDx using a structured, evidence-based approach, but there is very limited support for this in the literature – suggesting that many schools still leave the subject to individual precepting and mentoring in the context of case discussions. In this setting, educators may employ a variety of approaches, heuristics and mnemonic devices for generating differentials – likely including one or more of the MMTs evaluated in this study.

In the main, these traditional approaches are applied without explicit validation or rigorous supporting evidence. It is noteworthy, for example, that the VINDICATE(S) acrostic, also formulated as VITAMINCDE or even VITAMINSABCDEK [17,18,28], is one of the most commonly taught and commonly used MMTs for generating differentials by pathophysiology. Yet within the limitations of our study, it was shown to be one of the weakest and least popular of the techniques tested. In contrast, the *Mental CT Scan* was found to be both the most efficacious and the best-liked of the MMTs – at least as applied to cases involving anatomic diagnoses. Although forms of this visualization technique have been described sporadically in the literature [18,28,34], our adaptation of this anatomic visualization device – in which we ask students to mentally ‘scan’ a patient from front to back in the coronal projection, identifying each major tissue plane and compartment along the way – may be particularly attractive and useful to students as well as working clinicians. In any event, our findings suggest that this MMT should be given consideration as an explicitly taught technique for generating anatomic differentials.

The two remaining MMTs – *Constellations* and *Bundling* – were found in the study to be of intermediate popularity and equivalent (and possibly superior) to VINDICATES in terms of efficacy. These MMTs are interesting in the sense that they are not classic mnemonic ‘tricks’ for merely promoting recall, but instead are heuristics that stimulate pattern recognition – one of the key elements of diagnostic reasoning described in the literature [1,5,7]. The *Bundling* technique is a straightforward metacognitive cue to remind the student or clinician that – precisely as described in the DDx course – *‘diagnoses travel in packs.’* Thus, for a course case involving vertigo, the student might recognize that a differential including labyrinthitis also tends to include (at least initially) such entities as benign paroxysmal positional vertigo, Menière’s Disease, and vertebrobasilar insufficiency. In the study, students were not prepared with specific ‘bundles’ – they were simply taught the concept of diagnostic bundling, given a few examples, and provided with tips for recognizing bundles. Obviously, such an approach will be limited by the student’s knowledge base, which might account for its modest performance in this study. It may well be, however, that *Bundling* becomes more useful as a function of the clinician’s level of experience.

The *Constellations* technique resembles the method described by Sacher and Detsky [29], in that it bases pattern recognition on relatively small clusters of clinical information selected for high discriminating power. These need not be near-pathognomonic *pivots*, as described by Eddy and others [27,34,35], but should be findings that help to confine the diagnosis (e.g., in a case of abdominal pain, hematochezia will have more discriminating power than nausea). Our approach to this MMT appears to be novel, however, in the deliberate and explicit use of many different clusters of case data to produce sub-differentials, which can then be summed to generate one large ‘superset’ differential. As with *Bundling*, this MMT is likely to be most useful in experienced hands; nevertheless, it appeared to outperform all but the *Mental CT Scan* in terms of both efficacy and popularity. As such, it may represent a teachable form of pattern recognition, one that is more learner-friendly precisely because it offers the diagnostician multiple patterns to recognize.

Our study was constrained by certain limitations. In a yearlong protocol involving 114 students, the study was not sufficiently powered to detect small individual MMT effects; it is certainly possible that the benefit of individual MMTs, other than the *Mental CT Scan*, would achieve statistical significance in a larger study. Another significant limitation with respect to the assessment of individual MMTs is that each MMT was, in effect, assessed by a single pair of cases. Thus the per-MMT findings, while novel and intriguing, must be regarded as preliminary, pending larger studies focused on specific MMTs.

We were also concerned about potential variability within the A-B case pairs for each MMT. We believe our A-B/B-A crossover control design significantly diminished the likelihood that a given MMT’s effects were the result of inconsistencies in case difficulty. Nevertheless, our linear mixed-model analysis demonstrated small differences in cumulative performance based on the order (A-B vs. B-A) of case completion.

Our study design was such that all cases were completed in strictly timed 3-min windows, which introduced another potential limitation, i.e., that some of the MMTs might be less effective in such a time-limited setting. Some students commented, for example, that VINDICATES might be more useful if more time were allotted for its use. Perhaps this accounts for that MMT’s flat performance in the study, and this is a question that might be fruitfully addressed in future work. It might be argued, however, that a 3-min limit approximates a reasonable span of time for an experienced clinician, or even a well-prepared medical student, to generate an initial differential diagnosis, especially given the kind of time constraints under which physicians are increasingly expected to work. It is just these kinds of real-world considerations that make methods such as diagnostic checklists [19,22] or 2-D diagnostic grids [16] difficult to implement.

Also of interest is the question of whether the performance-enhancing effect of the DDx course and its MMTs is durable and persistent. The protocol described herein was not designed to address this question, but we hope to explore the matter in a future study. Informal feedback from fourth-year students and graduates has provided some cautious encouragement for the hypothesis that a formal, MMT-focused DDx course can help students to become better lifelong diagnosticians.

Within the context of our 3-phase DDx model (generation-filtration-ordering), this study was designed to assess techniques that operate primarily on the generation phase; that is, it is concerned with optimizing sensitivity to diagnostic possibilities. Whether this alone will support clinicians in making better final diagnoses is an open question; generation may be necessary for diagnosis, but it is not sufficient. Still, it may be considered a *sine qua non*, as well as a ‘bottleneck’ for many diagnosticians, particularly students – and the ‘failure to consider the correct diagnosis as a possibility’ [22] has been cited as the most common cause of diagnostic error. Moreover, it may be argued that the students’ use of the MMTs also promoted a degree of appropriate filtration, as significant improvements in differential size were generally observed in both screened and unscreened differentials throughout the study. This suggests that MMTs can increase diagnostic sensitivity without sacrificing specificity. In our ongoing studies, we are evaluating heuristics that more directly influence filtration (i.e., optimization of specificity) and ordering (i.e., optimized diagnostic priorities).

This study is, to our knowledge, the first to describe a conceptual, MMT-focused DDx course for medical students, and in this context, to develop preliminary data regarding comparative efficacy of commonly used, general-purpose MMTs for generating differential diagnoses. Our findings support the following guidance to medical educators who are motivated to accelerate the development of diagnostic skills in their students:

1. A formal, metacognition-focused DDx course, such as described herein, can enhance differential diagnosis generation skills beyond the traditional, case-oriented approach.
2. General-purpose differential diagnosis MMTs, whether taught formally or informally, are not all created equal, and emphasis should be given to those techniques best supported by evidence – noting, however, that there appears to be an aggregate or synergistic benefit to using MMTs together.
3. Medical students may indeed be good judges of which techniques and approaches – which ‘tricks’ – work best for them. Recognizing and embracing that is, perhaps, the real trick to teaching the art and science of differential diagnosis.
